# Specific expression of heme oxygenase-1 by myeloid cells modulates renal ischemia-reperfusion injury

**DOI:** 10.1038/s41598-017-00220-w

**Published:** 2017-03-15

**Authors:** Maxime Rossi, Antoine Thierry, Sandrine Delbauve, Nicolas Preyat, Miguel P. Soares, Thierry Roumeguère, Oberdan Leo, Véronique Flamand, Alain Le Moine, Jean-Michel Hougardy

**Affiliations:** 1 Institute for Medical Immunology (IMI), Université Libre de Bruxelles, Gosselies, Belgium; 2 Department of Urology, Hôpital Erasme, Université Libre de Bruxelles, Brussels, Belgium; 3 Department of Nephrology, Dialysis and Renal Transplantation, CHU de Poitiers, Poitiers, France; 4 Laboratory of Immunobiology, Institute for Molecular Biology and Medicine, Université Libre de Bruxelles, Gosselies, Belgium; 5 Instituto Gulbenkian de Ciência, Oeiras, Portugal; 6 Department of Nephrology, Dialysis and Renal Transplantation, Hôpital Erasme, Université Libre de Bruxelles, Brussels, Belgium

## Abstract

Renal ischemia-reperfusion injury (IRI) is a major risk factor for delayed graft function in renal transplantation. Compelling evidence exists that the stress-responsive enzyme, heme oxygenase-1 (HO-1) mediates protection against IRI. However, the role of myeloid HO-1 during IRI remains poorly characterized. Mice with myeloid-restricted deletion of HO-1 (HO-1^M-KO^), littermate (LT), and wild-type (WT) mice were subjected to renal IRI or sham procedures and sacrificed after 24 hours or 7 days. In comparison to LT, HO-1^M-KO^ exhibited significant renal histological damage, pro-inflammatory responses and oxidative stress 24 hours after reperfusion. HO-1^M-KO^ mice also displayed impaired tubular repair and increased renal fibrosis 7 days after IRI. In WT mice, HO-1 induction with hemin specifically upregulated HO-1 within the CD11b^+^ F4/80^lo^ subset of the renal myeloid cells. Prior administration of hemin to renal IRI was associated with significant increase of the renal HO-1^+^ CD11b^+^ F4/80^lo^ myeloid cells in comparison to control mice. In contrast, this hemin-mediated protection was abolished in HO-1^M-KO^ mice. In conclusion, myeloid HO-1 appears as a critical protective pathway against renal IRI and could be an interesting therapeutic target in renal transplantation.

## Introduction

Ischemia-reperfusion injury (IRI) is inherent to renal transplantation and leads to delayed graft function (DGF) of transplanted kidneys from deceased donors in up to 20 to 50% of cases^1, 2^. Later, DGF contributes to the reduced longevity of the kidney allografts, notably because of a higher risk of acute and chronic rejection^3, 4^. Basically, IRI is a two-phase phenomenon, including ischemia in the donor and reperfusion injury in the recipient. It combines major ischemia-induced cell stress, significant burst of free radicals, and intense inflammatory immune responses that lead to extensive cell injury, necrosis, and late interstitial fibrosis of the kidney allograft^1, 5^. Today, the increasing demand for renal allografts implies more frequent use of organs issued from extended criteria donors and this change leads to an increased risk of DGF^6, 7^. So far, there is no specific treatment of IRI and a better understanding of underlying mechanisms might lead to a better prevention of DGF and successful transplantations even with transplant from extended criteria donors.

Several natural cellular mechanisms can confer resistance against IRI, including the ubiquitous heme oxygenase (HO) cytoprotective pathway^8^. Upon cellular stress, the expression of HO isoform 1 (HO-1, encoded by *Hmox1*) is significantly induced. The main action of HO-1 is the enzymatic degradation of free heme, a source of reactive species massively released during IRI. HO-1 metabolizes free heme into carbon monoxide, biliverdin that can both provide resistance against IRI^8^. Interestingly, HO-1-deficient mice exhibit severe acute kidney injury (AKI) and death upon renal IRI^9, 10^. In humans, reduced capacity of the donor to express HO-1 was associated with poorer kidney allograft survival^11–13^. At the opposite, non-specific HO-1 induction with synthetic heme can confer significant resistance against renal IRI^14, 15^.

Myeloid cells (i.e. neutrophils, circulating monocyte, tissues macrophages and dendritic cells) can be important contributors to the pathogenesis of IRI-induced AKI^9, 16, 17^. During renal IRI, it has been shown that expression of HO-1 is an important trafficking regulator of myeloid cells^9^. Therefore, we hypothesized that the myeloid HO-1 might be critical in renal IRI. Herein, we compared IRI in transgenic mice with myeloid-restricted deletion of HO-1 (HO-1^M-KO^) to control mice. In addition, we used hemin to confer protection against IRI and to study the importance of a myeloid source of HO-1 in this preventive strategy.

## Materials and Methods

### Mice

HO-1^M-KO^ mice were generated using the Cre/loxP system as described^18^. Two transgenic lines were used for the myeloid-specific deletion of HO-1: (1) C57BL/6 *Hmox1* ^loxP/loxP^ mice in which *Hmox1* allele was flanked by *loxP* sites, and (2) C57BL/6 *LysM* ^Cre/wt^ mice in which Cre recombinase is under the control of a myeloid-restricted promoter (*LysM*, *lysozyme M*). HO-1^M-KO^ mice (i.e. *LysM* ^Cre/wt^ *Hmox1* ^loxP/loxP^) and littermate (LT) mice (i.e. *LysM* ^wt/wt^ *Hmox1* ^loxP/loxP^) were generated at the Institute for Medical Immunology by crossing C57BL/6 *Hmox1* ^loxP/loxP^ mice with C57BL/6 *LysM* ^Cre/WT^ mice. The *LysM* ^Cre^ transgene mediates a specific deletion of *loxP*-flanked *Hmox1* gene in myeloid cells. C57BL/6 wild-type (WT) mice were purchased from Harlan (Zeist, The Netherlands). LT or WT mice, when specified, were used as controls for HO-1^M-KO^ mice. Eight- to twelve-week-old male animals were used for all experiments, and animals were bred in our specific pathogen-free animal facility. All experiments were conducted in compliance with the Principles of Laboratory Animal Care formulated by the National Institute of Health (Guide for the Care and Use of Laboratory Animals, Eighth Edition, National Research Council, 2010) and were approved by the local committee for animal welfare (Commission d’éthique du Biopole ULB Charleroi).

### Renal IRI procedure

Mice were anesthetized with an intraperitoneal injection (340 μl/25 g) of a solution containing Dormicum^®^ (1 mg/ml; Roche), Fentanyl^®^ (78 μg/ml; Janssen-Cilag), and Haldol^®^ (5 mg/ml; Janssen-Cilag). Body temperature was maintained at 37 °C throughout the procedure. Kidneys were exposed through midline incision, and both renal pedicles were clamped for 26 minutes using nontraumatic microsurgical clamps (S&T Microsurgical Instruments). Evidence of ischemia was confirmed by visualizing dark color of clamped kidneys. Restoration of blood flow was monitored before closing incision. Sham-operated mice underwent the same procedure except for clamping of the pedicles. Mice were sacrificed 24 hours or 7 days after reperfusion and samples were collected. When specified, mice received an intraperitoneal injection of hemin (5 mg/kg) or saline 24 h prior to surgery.

### Preparation of hemin solution

Hemin (Ferriprotoporphyrin IX chloride, Sigma-Aldrich) was dissolved in 0.1 M NaOH, neutralized (to pH 7.2) with 1 M HCl and adjusted to concentration of 7.7 mM with distilled water. Aliquots were protected from light and stored at −80 °C until used. Hemin was then diluted in sterile saline (NaCl 0.9%) to appropriate concentration and filtered.

### Generation of bone marrow-derived macrophages (BMDMs) and culture

Bone marrow cells were isolated from femurs and tibias of WT, LT and HO-1^M-KO^ mice, and cultured in Petri dishes (Greiner Bio-one). Bone marrow cells were incubated at 37 °C in a 5% CO_2_ atmosphere. For generation of bone marrow-derived macrophages (BMDMs), bone marrow cells were grown in Dulbecco modified Eagle medium (DMEM) supplemented with L-Glutamine, 4.5 g/l glucose (Lonza), 10% heat-inactivated fetal calf serum (FCS), nonessential amino acids, sodium pyruvate, penicillin/streptomycin, β-mercaptoethanol, and 20% supernatant derived from macrophage colony-stimulating factor (M-CSF)-producing L929 cells. At day 3 of culture, 5 ml of complete medium containing 60% L929 cells supernatant was added to each dish. BMDMs were allowed to grow until day 7 after isolation. The purity of BMDMs in culture was over 97% as confirmed by a Fluorescence-activated cell sorting (FACS) analysis of CD11b and F4/80 expression. BMDMs were then collected and cultured in resting and stimulated conditions either with lipopolysaccharide (LPS) 100 ng/ml (Sigma-Aldrich) or hemin 15 μM (Sigma-Aldrich) by using complete medium supplemented with 2% L929 cells supernatant for 24 hours at 37 °C in a 5% CO_2_ atmosphere.

### RNA extraction and Real-Time Quantitative Reverse Transcription PCR (qRT-PCR)

Total RNA was extracted from BMDMs and kidney tissues using the MagnaPure LC RNA Isolation Kit High Performance and the MagnaPure LC RNA Isolation Kit III for tissue, respectively, according to manufacturer's instructions (Roche). Reverse transcription and real-time PCR were performed using LightCycler Multiplex RNA Virus Master (one-step procedure) on a LightCycler 480 apparatus (Roche). Glyceraldehyde 3-phosphate dehydrogenase (GAPDH) was used as a RNA loading control. The primers were custom ordered from Eurogentec as follows: GAPDH, forward, 5′-ATTGTCAGCAATGCATCCTG-3′, reverse, 5′-CCTTCCACAATGCCAAAGTT-3′ and probe, 5′-FAM-CCCTGGCCAAGGTCATCCATGA-TAMRA-3′; HO-1, forward, 5′-GCCGAGAATGCTGAGTTCAT-3′, reverse, 5′-AGGAAGCCATCACCAGCTTA-3′ and probe, 5′-FAM-AGAACTTTCAGAAGGGTCAGGTGTCCA-TAMRA-3′; IL-6, forward, 5′-GAGGATACCACTCCCAACAGACC-3′, reverse, 5′-AAGTGCATCATCGTTGTTCATACA-3′ and probe, 5′-FAM-CAGAATTGCCATTGCACAACTCTTTTCTCA-TAMRA-3′. Monocyte chemoattractant protein-1 (MCP-1), keratinocyte chemoattractant (KC), p53, and p21 primers were purchased as ready-made mix from Roche. BMDMs mRNA levels are expressed as 2^−ΔΔCT^ in which CT represents “cycle of threshold”, ΔΔCT = ΔCT_mouse of interest_ − ΔCT_naïve LT mouse_, and ΔCT = CT_gene of interest_ − CT_GAPDH_. Kidney tissue mRNA levels are expressed as 2^−ΔΔCT^ in which CT represents “cycle of threshold”, ΔΔCT = ΔCT_mouse of interest_ − ΔCT_sham-operated WT C57BL/6 mouse_, and ΔCT = CT_gene of interest_ − CT_GAPDH_.

### Western blot

Western blot analysis was performed as described^19^. BMDMs were lysed in radioimmunoprecipitation assay (RIPA) buffer (Sigma-Aldrich) containing protease inhibitor cocktail (Sigma-Aldrich). Protein concentration was measured with the Micro BCA Protein Assay Kit (Thermo Scientific). Primary antibodies used were Actin pAb (Sigma-Aldrich) and HO-1 mAb (clone EP1391Y, Abcam). Secondary antibody used was donkey anti-rabbit IgG- horseradish peroxidase (HRP) (GE Healthcare).

### Renal function assessment and histopathology

Renal function was evaluated by measuring creatinine in plasma samples as described^20^. Kidneys were fixed in 4% formaldehyde, embedded in paraffin, sectioned at 5-μm thickness, and stained with Periodic acid–Schiff–diastase and Masson Trichrome. Renal damage was assessed in a blinded manner by the Tubular Injury Score^21^. Briefly, the percentage of damaged tubules were assessed in the corticomedullary junction according to observation of necrosis, tubular dilatation, brush border loss, and cast deposition in 10 nonoverlapping fields for each sample (x400 magnification). A five-point scale was used: 0 = no damage, 1 = <10% of tubules injured, 2 = 10–25%, 3 = 25–50%, 4 = 50–75%, 5 = >75%. Interstitial fibrosis was quantified using NIH ImageJ software**.**

### Immunohistochemistry

Paraffin-embedded 5-µm kidney sections were deparaffinized and rehydrated. Endogenous peroxidase activity was first quenched by H_2_O_2_ peroxidase blocking reagent (DakoCytomation). Macrophages and neutrophils were detected by using an anti-F4/80 antibody (1:50; eBioscience) and an anti–Ly-6G antibody (1:50; BD Biosciences), respectively; for 30 minutes at room temperature (RT). Sections were then washed and incubated with a secondary biotinylated goat anti-rabbit antibody (1:500; Jackson Immunoresearch) for 30 minutes at RT. Streptavidin-HRP was added and coloration was revealed using diaminobenzidine (DAB) with the substrate chromogen system from Dakocytomation. The number of F4/80^+^ and Ly-6G^+^ cells was counted in 10 non-overlapping fields (x400 magnification). Nitrotyrosine staining was performed using an anti-nitrotyrosine antibody (1:400; Abcam) and the OptiView DAB IHC Detection Kit (Ventana Medical Systems) according to manufacturer’s instructions. Nitrotyrosine intensity in the renal cortex was quantified using NIH ImageJ software.

### Kidney homogenates preparation and Enzyme-Linked Immunosorbent Assay

Kidneys were diluted in lysis solution containing RIPA buffer (Sigma-Aldrich), protease inhibitor cocktail (Sigma-Aldrich), and phosphatase inhibitor cocktail PhosSTOP (Roche Life Science). Kidneys were homogenized with the MagNa Lyser (Roche Diagnostics). Homogenates were subsequently centrifuged at 12,000 rpm for 20 minutes at 4 °C, and the supernatants were stored at −80 °C until use. HO-1 and p62 enzyme-linked immunosorbent assay (ELISA) kits were purchased from Enzo Life Sciences. Interleukin-6 (IL-6), KC, and MCP-1 ELISA kits were purchased from R&D Systems. Renal cytokines were measured according to manufacturer's instructions. Values were corrected for the amount of renal proteins using Micro BCA Protein Assay Kit (Thermo Scientific).

### Kidney tissue digestion protocol for flow cytometry

Kidneys were harvested and washed in RPMI 1640 medium supplemented with L-Glutamine and 25 mM Hepes (Lonza). Kidneys were dissociated in 5 ml digestion buffer containing collagenase IV (Worthington Biochemical) and DNase I (Roche) using the mouse lung dissociation program 1 on gentleMACS Dissociator (Miltenyi Biotec). Then, samples were incubated for 20 minutes at 37 °C and agitated every 15 minutes. Complete tissue dissociation was achieved using the mouse spleen dissociation program 4 on gentleMACS Dissociator (Miltenyi Biotec). Cell suspension was passed through a 70 μm cell strainer (BD Biosciences) and was adjusted to a 30 ml volume with Phosphate Buffer Saline (PBS)/Bovine Serum Albumin (BSA) 0.5%/Ethylenediaminetetraacetic acid (EDTA) 2 mM. Samples were centrifuged at 300 g for 5 minutes at 4 °C. Cell pellets were resuspended in 1 ml PBS-BSA 0.5%-EDTA 2 mM and suitable for flow cytometry staining protocol.

### Flow cytometry

Kidneys were prepared as described above. HO-1 intracytoplasmic staining was performed through indirect labeling after cell surface marker staining using the Intracellular Fixation and Permeabilization Buffer Set (eBioscience) according to manufacturer’s instructions. PE-conjugated anti-mouse F4/80 (clone BM8), and anti-mouse CD16/CD32 (Fc block, clone 93) were purchased from eBioscience. FITC-conjugated anti-mouse IgG1 (clone A85-1), and APC-Cy7-conjugated anti-mouse CD11b (clone M1/70) were purchased from BD Biosciences. Unconjugated anti-mouse HO-1 (clone HO-1-1), and PE/Cy7-conjugated anti-mouse CD45 (clone 30-F11) were purchased from Abcam and BioLegend, respectively. Cytometric analysis was performed on a BD LSRFortessa cell analyzer (BD Biosciences) using FlowJo software (FlowJo LLC).

### Statistical analysis

All data are expressed as mean ± standard error of the mean (SEM). A two-tailed nonparametric Mann-Whitney *U* test was used; *P*-values < 0.05 were considered to represent statistical significance. All graphs and statistical analyses were performed using GraphPad Prism 6.00 for Mac OS X (GraphPad Software, La Jolla California USA, www.graphpad.com).

## Results

### Renal myeloid cells upregulate HO-1 upon renal IRI

**Figure 1:**
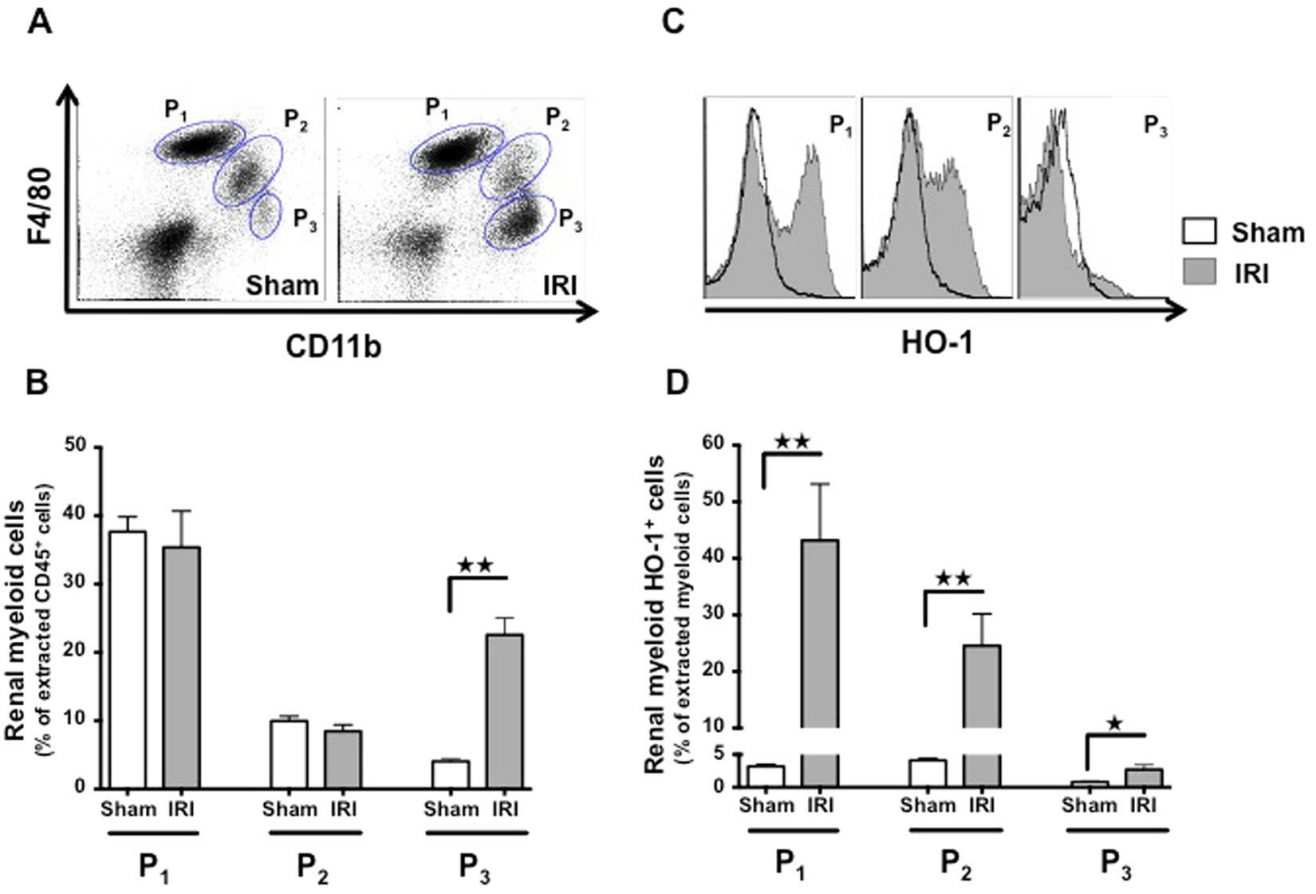
IRI induces HO-1 expression by kidney-infiltrating myeloid cells. Wild-type mice (WT) underwent either bilateral renal IRI for 26 minutes or sham surgery. Twenty-four hours later, mice were sacrificed and kidneys were harvested and processed for flow cytometry analysis. (**A**) Representative dot plots of sham-operated and IRI mice demonstrating three myeloid-cell type populations in the kidney. These distinct populations were CD11b^−^ F4/80^+^ (P_1_), CD11b^+^ F4/80^lo^ (P_2_), and CD11b^hi^ F4/80^−^ (P_3_). (**B**) Quantification of renal myeloid cells, presented as a proportion of the CD45^+^ cells extracted from the kidney after IRI (grey bars) or sham surgery (white bars). (**C**) Representative histograms depicting the level of intracellular HO-1 expression in the myeloid populations after IRI (grey filled area) or sham surgery (bold curve area). (**D**) Quantification of renal myeloid HO-1^+^ cells, presented as a proportion of the myeloid cells extracted from the kidney after IRI (grey bars) or sham surgery (white bars). Results are expressed as the mean ± SEM, ^★^p < 0.05; ^★★^p < 0.01. n = 5–7 per group.

Based on CD11b and F4/80 expression by flow cytometric analysis, we defined three major myeloid cell sub-populations in the kidney (Fig. 1A). These populations were CD11b^−^ F4/80^+^ (defined as P_1_), CD11b^+^ F4/80^lo^ (defined as P_2_) and CD11b^hi^ F4/80^−^ cells (defined as P_3_). Most of the P_3_ cells were neutrophils according to their high Ly6G expression (Supplemental Fig. S1). Upon renal IRI, we noted a significant increase in the number of P_3_ cells, while P_1_ and P_2_ subsets remained unaffected (Fig. 1A,B). Interestingly, we observed a significant increase in HO-1 expression within both P_1_ and P_2_ populations 24 hours after reperfusion whereas P_3_ cells only displayed a slight induction of HO-1 (Fig. 1C,D).

### Myeloid HO-1 protects kidney from IRI

**Figure 2:**
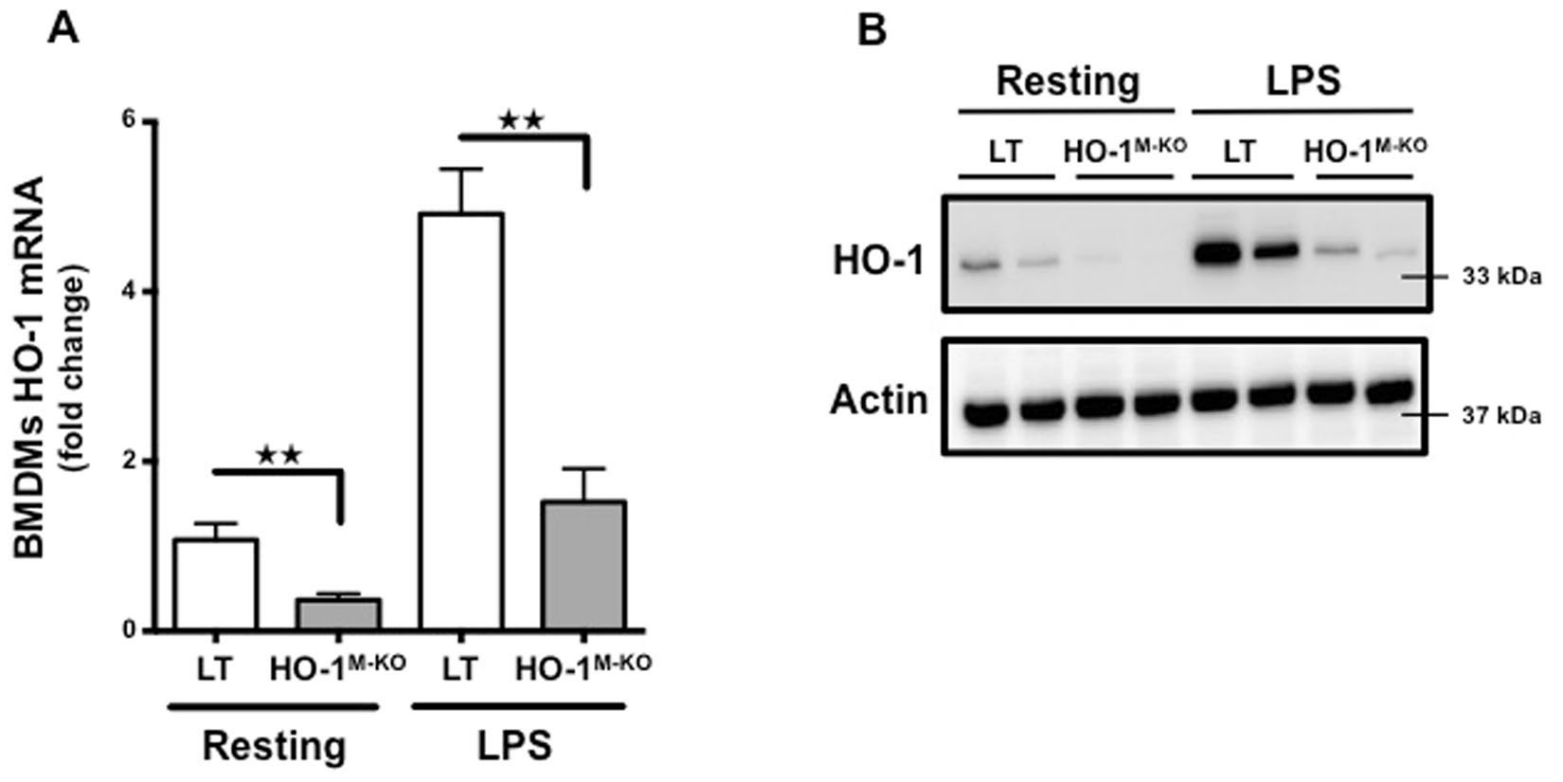
Myeloid-restricted deletion of HO-1 in HO-1^M-KO^ mice. (**A**) qRT-PCR analysis of HO-1 mRNA levels and (**B**) representative images of western blot analysis for HO-1 in BMDMs generated from LT (white bars) and HO-1^M-KO^ (grey bars) mice in resting and after LPS stimulation (100 ng/ml) for 24 hours. Results are expressed as the mean ± SEM, ^★★^p < 0.01 and representative of at least 3 independent experiments. n = 5 per group for HO-1 mRNA levels.

**Figure 3:**
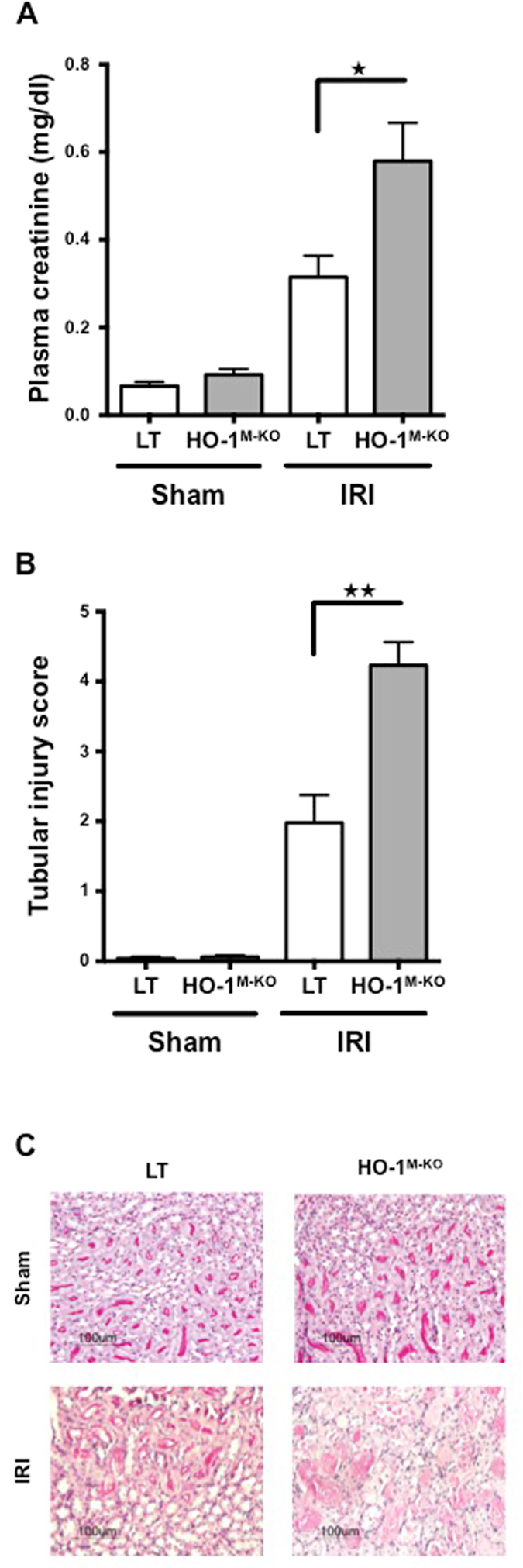
Myeloid-restricted ablation of HO-1 exacerbates renal IRI. (**A**) Plasma creatinine levels and (**B**) histological scoring for tubular damage in LT (white bars) and HO-1^M-KO^ (grey bars) mice subjected to sham surgery or 24 hours of reperfusion after renal IRI. Results are expressed as the mean ± SEM, ^★^p < 0.05; ^★★^p < 0.01. n = 7–9 for IRI groups and n = 5 for sham groups. (**C**) Representative sections of corticomedullary junction from sham-operated and IRI after 1 day of reperfusion in LT and HO-1^M-KO^ mice (PAS-D stained). Magnification, x200. Scale bar, 100 μm.

**Figure 4:**
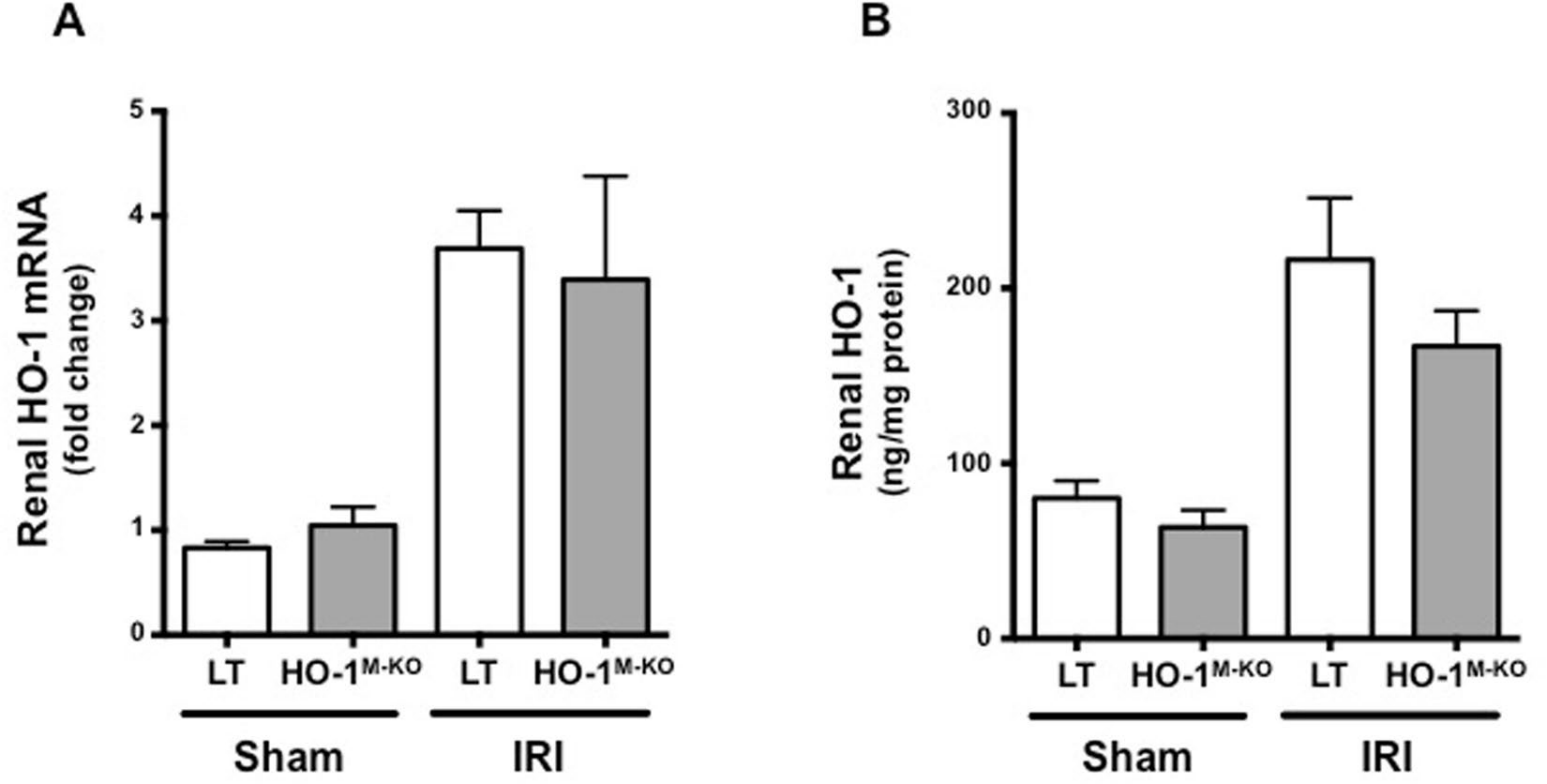
Whole-tissue renal expression of HO-1 is similar between in LT and HO-1^M-KO^ mice. (**A**) qRT-PCR analysis of HO-1 mRNA levels and (**B**) ELISA for HO-1 in LT (white bars) and HO-1^M-KO^ (grey bars) mice subjected to sham surgery or 24 hours of reperfusion after renal IRI. Results are expressed as the mean ± SEM. n = 5–8 for IRI groups and n = 5 for sham groups.

HO-1^M-KO^ mice were used to determine the role of myeloid HO-1 during IRI. Myeloid-restricted deletion of HO-1 was confirmed in both resting and LPS-stimulated BMDMs (Fig. 2A,B). We identified that HO-1^M-KO^ mice exhibit increased susceptibility to renal IRI as demonstrated by the significant loss of renal function and more severe tubular injuries 24 hours after renal IRI in comparison to LT mice (Fig. 3A–C). Interestingly, after renal IRI, HO-1 expression within the whole kidney was identical between LT and HO-1^M-KO^ mice (Fig. 4A,B), suggesting that, despite representing a minor source of HO-1 from a quantitative point of view, myeloid HO-1 mediates significant renal protection during IRI.

### Myeloid HO-1 mitigates both innate immune responses and oxidative stress upon renal IRI

**Figure 5:**
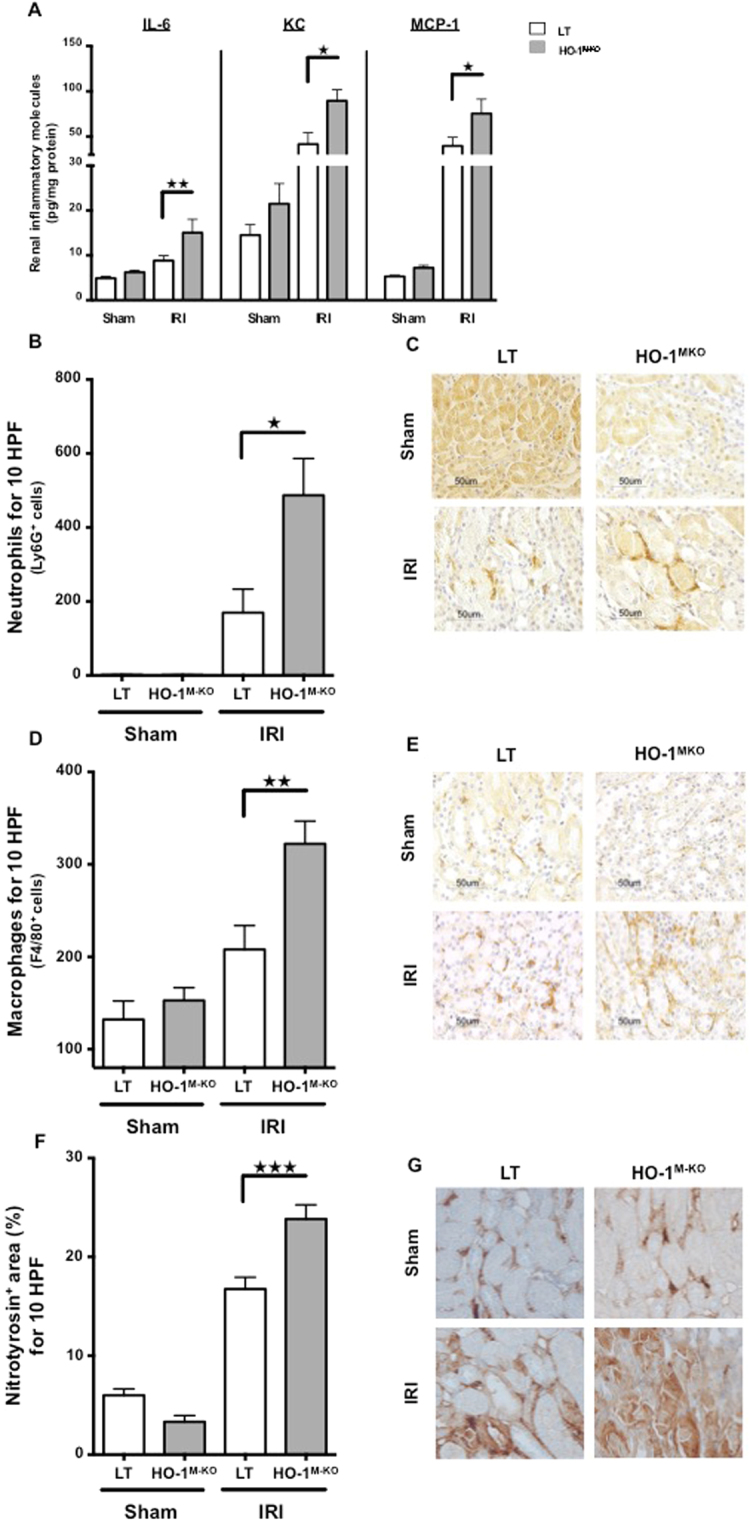
Myeloid HO-1 dampens innate immune responses and oxidative stress upon renal IRI. LT (white bars) and HO-1^M-KO^ mice (grey bars) underwent sham surgery or 1 day of reperfusion after IRI. (**A**) Proinflammatory mediator levels in kidney. (**B**) Neutrophil and (**D**) macrophage influx counts. Representative renal sections stained for Ly6G (**C**) and F4/80 (**E**). Magnification, x400. Scale bar, 50 μm. (**F**) Percentage of nitrotyrosine-positive area in corticomedullary junction and (**G**) representative sections of nitrotyrosine immunostaining. Magnification, x400. Quantification for nitrotyrosine was done by using ImageJ software. Results are expressed as the mean ± SEM, ^★^p < 0.05; ^★★^p < 0.01; ^★★★^p < 0.001. n = 7–9 for IRI groups and n = 5 for sham groups.

The impact of myeloid HO-1 on renal inflammation 24 hours after IRI was analyzed. HO-1^M-KO^ mice expressed increased levels of renal inflammatory mediators (i.e. IL-6, KC, and MCP-1) in comparison to LT (Fig. 5A). In addition, HO-1^M-KO^ mice also exhibited increased neutrophil and macrophage infiltrates surrounding necrotic tubular cells in the corticomedullary junction (Fig. 5B–E). Finally, accumulation of tubular nitrotyrosine, a well-described marker of oxidative damage^22^, was exacerbated within the renal cortex of HO-1^M-KO^ mice in comparison to LT (Fig. 5F,G).

### Myeloid HO-1 deficiency leads to impaired tubular repair and renal interstitial fibrosis

**Figure 6:**
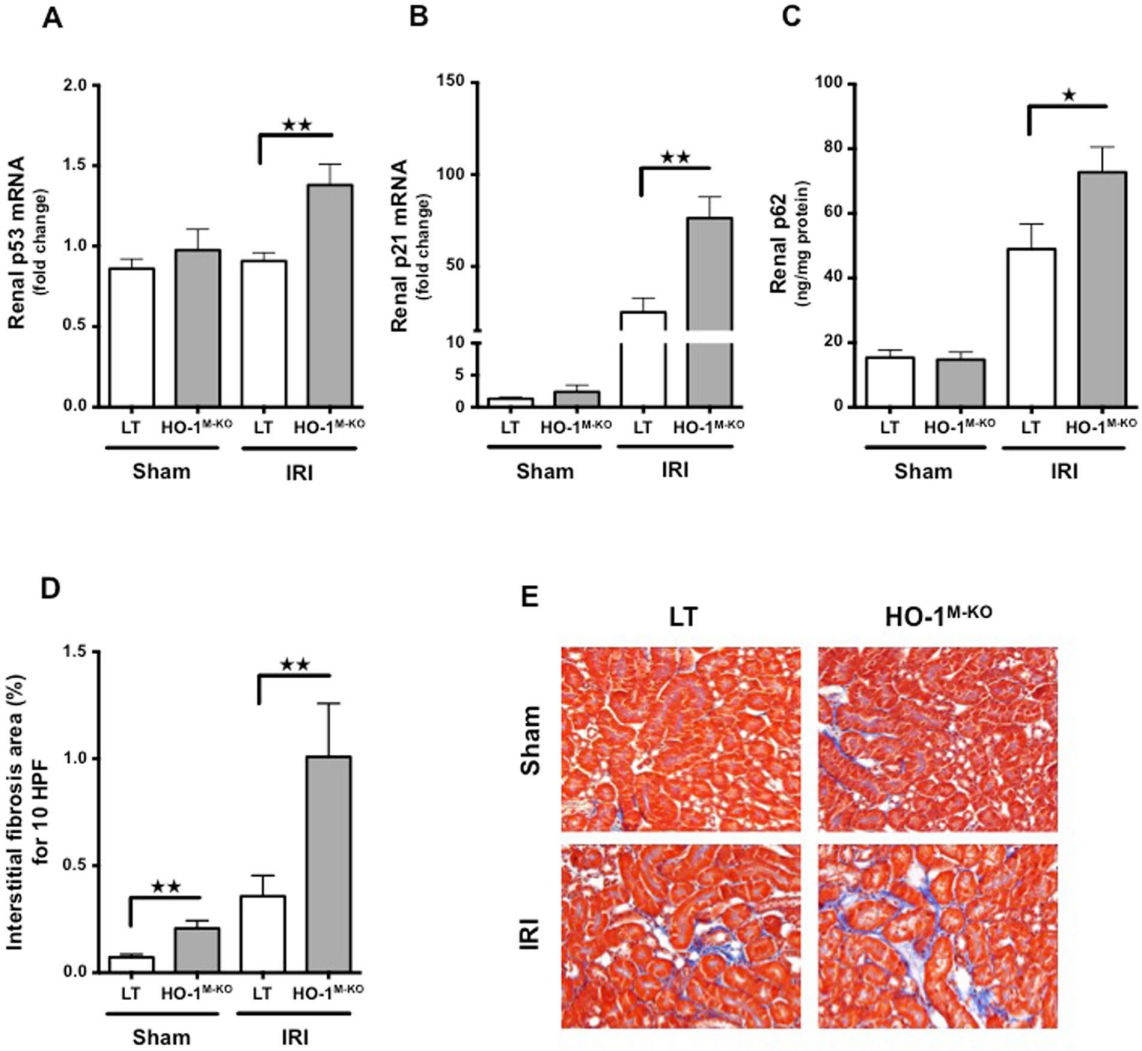
Myeloid HO-1 promotes renal repair and attenuates interstitial fibrosis induced by renal IRI. (**A**) p53 mRNA, (**B**) p21 mRNA and (**C**) p62 renal expression in LT (white bars) and HO-1^M-KO^ (grey bars) mice subjected to sham surgery, 4 hours (p53 mRNA analysis) or 24 hours (p21 mRNA and p62 analysis) of reperfusion after renal IRI. Results are expressed as the mean ± SEM, ^★^p < 0.05; ^★★^p < 0.01. In p53/p21 experiments, n = 6–8 for IRI groups and n = 5–6 for sham groups. In p62 experiment, n = 9–10 for IRI groups and n = 6 for sham groups. (**D**) Percentage of fibrosis area and (**E**) representative renal sections of Masson Trichrome staining from sham-operated and IRI after 7 days of reperfusion in LT (white bars) and HO-1^M-KO^ (grey bars) mice. Quantification was done using ImageJ software. Results are expressed as the mean ± SEM, ^★★^p < 0.01. n = 3–4 for IRI groups and n = 5 for sham groups. Magnification, x200.

Tubular cell-cycle arrest represents a major event that promotes interstitial fibrosis and finally chronic kidney disease (CKD) upon IRI^5, 23^. In order to investigate a possible role of myeloid HO-1 in the prevention of CKD, we analyzed the expression of the cell-cycle inhibitor p53 and its target p21. As compared to LT, HO-1^M-KO^ mice exhibited a significant increase in both p53 and p21 mRNA expression 4 hours and 24 hours after IRI, respectively (Fig. 6A,B). In addition, we noted a significant accumulation of renal p62 24 hours post IRI in the HO-1^M-KO^ animals as compared to LT, suggesting potential impaired autophagy, a phenomenon known to enhance interstitial fibrosis upon tubular stress^24^ (Fig. 6C). Finally, 7 days after reperfusion, HO-1^M-KO^ mice exhibited more renal interstitial fibrosis in comparison to LT (Fig. 6D,E).

### Hemin-mediated protection against IRI depends on HO-1 upregulation by renal CD11b^+^F4/80^lo^ myeloid cells

**Figure 7:**
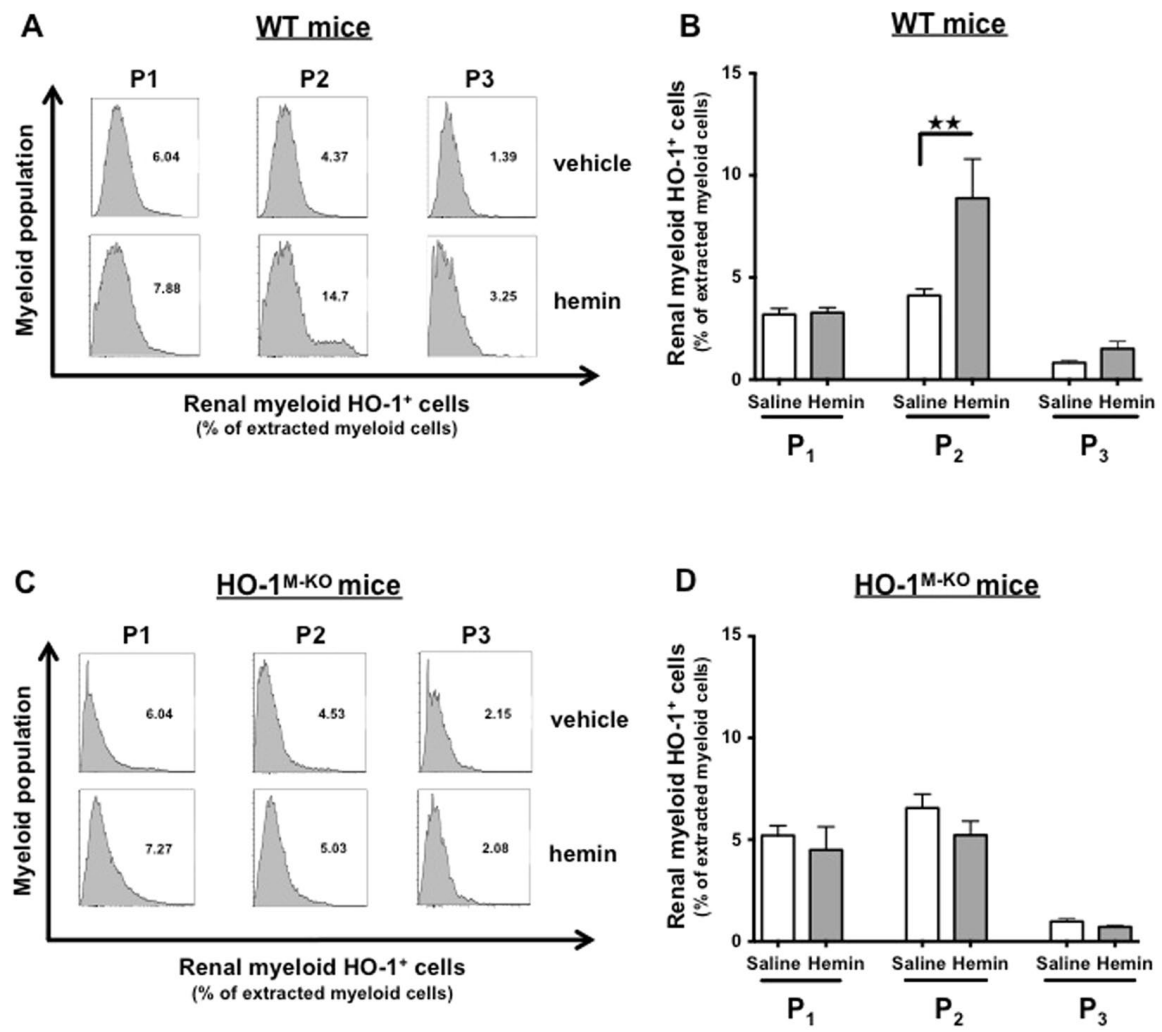
Hemin specifically induces HO-1 expression in renal CD11b^+^ F4/80^lo^ (P_2_) myeloid cells. WT (**A**,**B**) and HO-1^M-KO^ mice (**C**,**D**) were treated with hemin (5 mg/kg) or saline. Twenty-four hours after intraperitoneal injection, kidneys were harvested and homogenized for flow cytometry analysis. The renal myeloid cell populations were characterized according to the expression of CD11b and F4/80 surface markers (i.e., CD11b^−^ F4/80^+^ (P_1_), CD11b^+^ F4/80^lo^ (P_2_), and CD11b^hi^ F4/80^-^(P_3_)). (**A**,**C**) Representative histograms depicting the level of intracellular HO-1 expression in the myeloid subpopulations after hemin administration or saline injection. (**B**,**D**) Quantification of renal myeloid HO-1^+^ cells, presented as a proportion of the myeloid cells extracted from the kidney after hemin (grey bars) or saline (white bars). Results are expressed as the mean ± SEM, ^★★^p < 0.01. n = 7–8 per group in WT mice and n = 5 per group in HO-1^M-KO^ mice.

**Figure 8:**
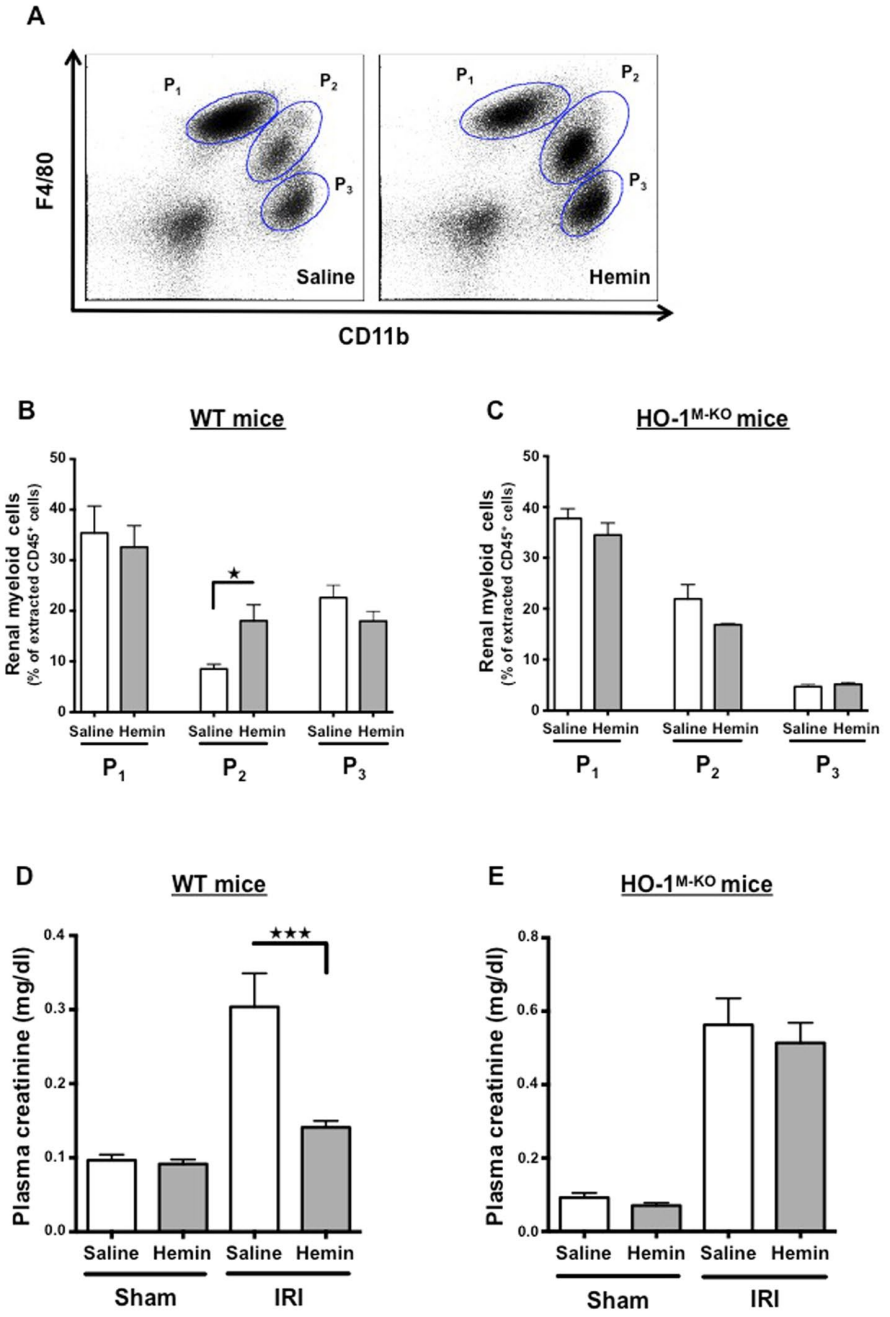
Renal CD11b^+^ F4/80^lo^ (P_2_) myeloid cells modulate myeloid HO-1-mediated protection against IRI. WT and HO-1^M-KO^ mice were treated with hemin (5 mg/kg, grey bars) or saline (white bars) 24 hours before renal IRI. At day 1 of reperfusion, mice were sacrificed. (**A**) Representative dot plots of saline- and hemin-treated WT IRI mice showing the myeloid cell populations in the kidney according to the expression of CD11b and F4/80 surface markers (i.e., CD11b^−^ F4/80^+^ (P_1_), CD11b^+^ F4/80^lo^ (P_2_), and CD11b^hi^ F4/80^−^(P_3_)). (**B**) Quantification of renal myeloid cells upon IRI, presented as a proportion of the renal CD45^+^ cells extracted from saline- and hemin-treated WT IRI mice. Results are expressed as the mean ± SEM, ^★^p < 0.05. n = 5–6 per group in WT mice. (**C**) Quantification of renal myeloid cells upon IRI, presented as a proportion of the renal CD45^+^ cells extracted from saline- and hemin-treated HO-1^M-KO^ IRI mice. Results are expressed as the mean ± SEM. n = 3–5 per group in HO-1^M-KO^ mice. Plasma creatinine levels in saline- and hemin-treated WT (**D**) and HO-1^M-KO^ (**E**) mice subjected to sham surgery or 24 hours of reperfusion after renal IRI. Results are expressed as the mean ± SEM, ^★★★^p < 0.001. In WT mice, n = 16–20 for IRI groups and n = 6 for sham groups. In HO-1^M-KO^ mice, n = 9 for IRI groups and n = 5 for sham groups.

To determine whether the protection against IRI conferred by hemin pretreatment involved HO-1^+^ myeloid cells, we treated HO-1^M-KO^ and WT mice either with hemin (5 mg/kg) or saline. Twenty-four hours after hemin administration, WT mice exhibited strong HO-1 expression within the renal P_2_ cells, while neither the P_1_ nor P_3_ subsets increased their HO-1 expression (Fig. 7A,B). Interestingly, hemin also induced HO-1 expression within the same subset of splenic myeloid cells (i.e. P_2_) (Supplemental Fig. S2). In contrast, no HO-1 induction in renal myeloid cells from HO-1^M-KO^ mice was noted (Fig. 7C,D). Twenty-four hours after renal IRI, the proportion of HO-1^+^ P_2_ cells increased in the hemin- vs. the saline-treated WT mice while it remained unchanged in HO-1^M-KO^ mice in both conditions (Fig. 8A–C). Concurrently, hemin-treated WT mice displayed renal resistance against IRI (Fig. 8D, Supplemental Fig. S3A–C) while hemin did not preserve renal function in HO-1^M-KO^ mice (Fig. 8E). These results strongly suggest that CD11b^+^ F4/80^lo^ cells (i.e. P_2_) are the main protective myeloid source of HO-1 within the kidney upon IRI. We also observed that, in comparison to WT mice, LT did not benefit from the protection against renal IRI when they were treated with hemin 24 hours before IRI (Supplemental Fig. S4A). This could be explained by a hypomorphic HO-1 allele in LT that was due to the modification of the *Hmox1* allele with *loxP* sites (Supplemental Fig. S4B–D). However, the HO-1 hypomorphism was not sufficient to provoke particular susceptibility to renal IRI in LT as attested by similar creatinine levels compared to WT mice (Supplemental Fig. S5).

## Discussion

We have shown that myeloid HO-1 mediates protection against renal IRI and that its preemptive induction by hemin confers significant resistance against renal IRI. These findings contrast with the common belief that only epithelial (i.e. tubular cells) and endothelial cells are the critical source of HO-1 during renal IRI. This hypothesis was mainly supported by the intense susceptibility of fully HO-1-deficient to renal IRI^10, 25, 26^. However, Ferenbach DA *et al.* already demonstrated that genetically modified or hemin-induced HO-1^+^ macrophages, a subset of myeloid cells, mediate protection against renal IRI^27, 28^. Our results strongly support this observation. In addition to these previous studies, we have observed that, in response to IRI, naturally occurring myeloid HO-1 may already modulate the severity of AKI. Indeed, even if we showed that the global expression of HO-1 in the whole kidney was not affected, the absence of myeloid HO-1 was critical in the outcome of renal IRI. More recently, Hull *et al.* showed that HO-1 is a critical regulator of the trafficking of myeloid cells in AKI. However they did not report a difference between LT and HO-1^M-KO^ mice in term of renal function (i.e. plasma creatinine) or in tubular damage 1 day after reperfusion^9^. In contrast, our results pointed out the myeloid HO-1 as a critical regulator of the earliest phases of IRI (i.e. lower plasma creatinine, tubular damage, and renal inflammation) that may mitigate the risk of severe AKI upon IRI. By inference, as severe IRI in a renal transplant also increases its immunogenicity, we may postulate that, by downsizing the early inflammatory response, the induction of myeloid HO-1 could be a regulatory mechanism decreasing transplant alloreactivity.

A cell-cycle arrest at the G2/M phase is associated with maladaptive repair and subsequent fibrosis in renal IRI^5, 23^. In our experiments, HO-1^M-KO^ mice exhibited impaired renal repair upon IRI as suggested by the upregulation of cell-cycle regulatory proteins (i.e. p53, p21), potential disruption of autophagy (i.e. p62 accumulation) and early interstitial fibrosis, a central marker of CKD. The roles of cell-cycle inhibitors p53/p21 in the pathogenesis of AKI are complex and remain a matter of debate. For instance, if p53 expression by leukocytes has been identified as protective against AKI, its induction in tubular cells was associated with worsened AKI and subsequent interstitial fibrosis^29–31^. In line with a previous study^32^, the cell-cycle inhibitor p21 has been shown protective in AKI by mediating a cell-cycle arrest in G1 phase and therefore allowing repair of DNA-damage^33^. However, the authors observed that p21 was not involved in protection against renal fibrosis^33^. Herein, we focus on the link between p21 and the progression of AKI to fibrosis. Interestingly, even if p21 expression may confer early resistance to AKI, p21 expression by tubular cells also reflects their cellular senescence that is associated with limited regenerative ability of tubular cells and interstitial fibrosis following AKI suggesting an additional role for p21^34, 35^. Indeed, these different data are consistent with the recent concept that p21 has different effects during AKI or CKD/fibrosis progression^36^.

Altogether, these observations sustain the hypothesis that the upregulation of both p53 and p21 upon IRI in HO-1^M-KO^ kidneys may encourage fibrotic processes in HO-1^M-KO^ mice. Interestingly, fully HO-1-deficient mice exhibit more severe interstitial fibrosis upon AKI^37^ and renal fibrosis was also reported in HO-1^M-KO^ mice 1week after IRI^9^. Further, our results suggest a link between myeloid HO-1 deficiency and renal fibrosis because of maladaptive repair.

Even if the role of myeloid HO-1 as a critical modulator of the late fibrotic processes remains a matter of debate, it has been shown that AKI could lead to severe CKD. Indeed, this is supported by the recent key concept of cell-cycle arrest and maladaptive repair of tubular cells that is induced by AKI itself^5, 23^. Briefly, a clear correlation between the severity of the renal lesions and the risk of maladaptive repair has been established in several experimental models, leading to interstitial fibrosis and subsequent CKD^5, 23^. Even if the loss of myeloid HO-1 expression before IRI was responsible for the observed kidney injury, a specific role for HO-1 during the later phases of renal repair after IRI is not excluded. For instance, HO-1 may decrease TGF-β expression at the tubular level, thereby preventing renal fibrosis^38^. HO-1 was also shown to modulate tubular autophagy, a major event involved in cell repair after insult^39^. Of interest, we have observed that HO-1^M-KO^ mice exhibited p62 accumulation upon renal IRI which may be seen as a surrogate marker of impaired autophagy^40^.

Specific deletion of myeloid HO-1 was associated with sustained oxidative damage within the corticomedullary junction upon IRI. It is well known that HO-1 limits the heme- and hydrogen peroxide-mediated oxidative stress that heavily damages DNA and proteins and leads to cell death^41^. However, the fact that HO-1^M-KO^ mice exhibit intense tubular damage due to excessive oxidative stress strongly suggests potential cross-talk between the tubular environment and the HO-1^+^ myeloid cells. Accordingly, the release of by-products of heme degradation by HO-1^+^ myeloid cells (i.e. carbon-monoxide, IL-10) may inhibit the apoptosis of the surrounding tubular cells, thereby promoting their survival^42^. Interestingly, HO-1^M-KO^ mice exhibited sustained p53 expression in the whole kidney upon IRI. As tubular cells are major components of kidney extract, we may hypothesize that myeloid HO-1 may regulate some of their important functions upon IRI, decreasing the risk of both tubular apoptosis and cell-cycle arrest.

It has been shown that HO-1 expression was associated with CD11b^+^ F4/80^lo^ macrophages that exhibit regulatory properties (i.e. “M2” macrophages)^43^. These M2 macrophages are potential modulators of inflammatory responses induced by renal IRI, thereby promoting renal repair after insult^16^. In this condition, HO-1^+^ myeloid cells may promote the development of a microenvironment dominated by regulatory macrophages that efficiently dampen early inflammatory responses and delay the onset of interstitial fibrosis^44^. However, the molecular mechanism of macrophage polarization mediated by HO-1 remains to be elucidated^44^. Interestingly, HO-1 modulates IL-10 expression^45, 46^ and HO-1^+^ macrophages express increased level of IL-10^27^ suggesting a positive-feed-forward loop between these two anti-inflammatory factors, favoring the acquisition of an IL-10-producing, M2-like phenotype. The macrophage-derived molecules that promote kidney repair are poorly known^47^. However, both the macrophage-secreted Wnt7b and chitinase-like protein BRP-39 have been shown to promote kidney regeneration after IRI^48, 49^. Accordingly, we may hypothesize that our HO-1^+^ CD11b^+^ F4/80^lo^ anti-inflammatory macrophages protect the renal parenchyma upon IRI by secretion of some mediators which may interact with renal epithelial cells.

Even if we did not demonstrate it in our experiments, the intense inflammatory response that we observed in HO-1^M-KO^ kidneys upon IRI might be explained through a phenotypic polarization toward “M1” macrophages. Indeed, in absence of HO-1, a lack of M2 macrophages was observed with an excess of inflammatory “M1” macrophages^50^. Then, these macrophages secrete pro-inflammatory mediators that amplify intrarenal inflammation and injury through interaction with kidney resident cells^16, 47^. Therefore, we assume that HO-1, in addition to its protective role against oxidative stress at the cellular level, also plays a critical role in the regulation of early innate immune responses induced by renal IRI.

The origin of HO-1^+^ myeloid cells that protect the injured kidney remains largely unknown. Theoretically, renoprotection might be provided by both resident and infiltrating HO-1^+^ myeloid cells in the kidney. In line with previous report^28^, we showed that, even in absence of IRI, hemin upregulated the HO-1 expression within CD11b^+^ F4/80^lo^ myeloid cells in the kidney. This observation suggests that tissue-resident myeloid cells might be involved in the earliest phase of renal IRI. The recruitment of splenic macrophages that protect against IRI was already observed^51, 52^. We also noted that hemin induced HO-1 within spleen CD11b^+^ F4/80^lo^ myeloid cells suggesting that extra-renal HO-1^+^ myeloid cells might represent a reservoir that can be recruited in the injured kidney after renal IRI. Indeed, one day after reperfusion, we observed a higher proportion of CD11b^+^ F4/80^lo^ myeloid cells within the kidney of hemin-treated WT mice suggesting that these protective cells could have been recruited from extra-renal sites such as the spleen.

We identified that the intensity of myeloid HO-1 expression was an important determinant of efficient hemin-mediated protection against renal IRI. Indeed, we found that the floxation of the *Hmox1* allele with *loxP* sites in LT significantly decreased both native and induced HO-1 expression in comparison to WT mice. This “hypomorphism” induced by allele floxation has been described in others *Cre-LoxP* knockout systems^53, 54^. Interestingly, even if the HO-1 hypomorphism was insufficient to cause particular susceptibility to renal IRI in LT compared to WT mice, it leaded to the loss of the hemin-mediated protection in LT. Therefore, we may postulate that myeloid HO-1 expression induced by hemin requires a critical level to be effective and that this minimal threshold in HO-1 expression was not reached in LT. By inference, the length polymorphism of guanosine thymidine dinucleotide (GT)_n_ repeats in the promoter region of *Hmox1* was associated with lower HO-1 activity^55^, mimicking the effect of a hypomorphic allele. Interestingly, longer (GT)_n_ repeats in the *Hmox1* promoter were associated with the occurrence of CKD and decreased renal function after cardiac surgery or kidney transplantation in humans^11, 56–58^. Also, it has been largely reported that hemin preconditioning significantly mitigates IRI-induced AKI^14, 15^. A recent study showed that hemin safely induces HO-1 in deceased donor renal transplant recipients but the benefit of this approach on the renal allograft survival remains still unknown^59^. Interestingly, myeloid HO-1 has been involved in the suppression of alloreactivity^60–62^. This suggests that myeloid HO-1 could have additional regulatory properties that may contribute to both better survival and tolerance of the allograft after IRI. However, further studies are needed to confirm the benefit of the HO-1 induction in renal transplantation.

In conclusion, our results strongly support the importance of HO-1^+^ myeloid cells for conferring resistance against renal IRI and are in line with other recent published works^9, 27, 28^. Myeloid HO-1 appears as a critical modulator of the interstitial inflammation and subsequent fibrosis that both follow renal IRI. Pharmacological HO-1 induction by hemin before renal IRI could be an efficient preventive strategy for limiting kidney damage in many situations such as renal transplantation.

## Electronic supplementary material

Supplementary information
