# Supplementary material for: Specific expression of heme oxygenase-1 by myeloid cells modulates renal ischemia-reperfusion injury
Source: Sci Rep. 2017 Mar 15;7:197. doi: 10.1038/s41598-017-00220-w (PMC5428056; doi:10.1038/s41598-017-00220-w)
Supplement: Supplementary file 1 — Supplementary information [file 41598_2017_220_MOESM1_ESM.pdf]

Specific expression of heme oxygenase-1 by myeloid cells modulates renal ischemia-reperfusion injury

Maxime Rossi, Antoine Thierry, Sandrine Delbauve, Nicolas Preyat, Miguel P. Soares, Thierry Roumeguère, Oberdan Leo, Véronique Flamand, Alain Le Moine and Jean-Michel Hougardy

Supplementary Information

Supplemental Fig. S1. Ly6G expression within renal myeloid cells

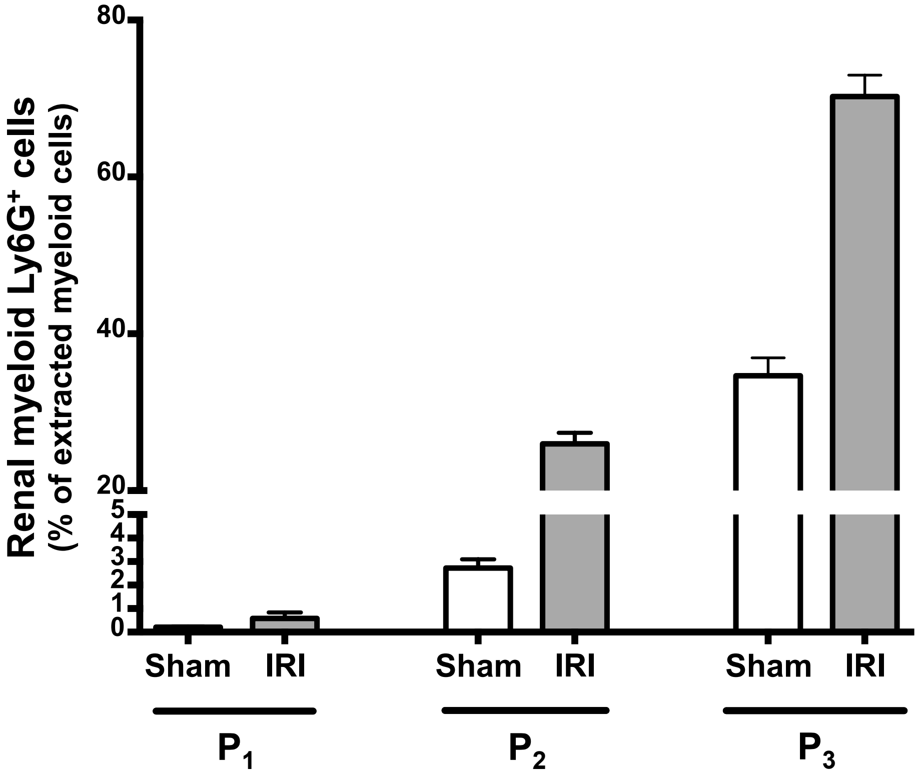

Supplemental Fig. S2. Hemin induces HO-1 expression in splenic CD11b$^+$ F4/80^{lo} (P_2) myeloid cells.

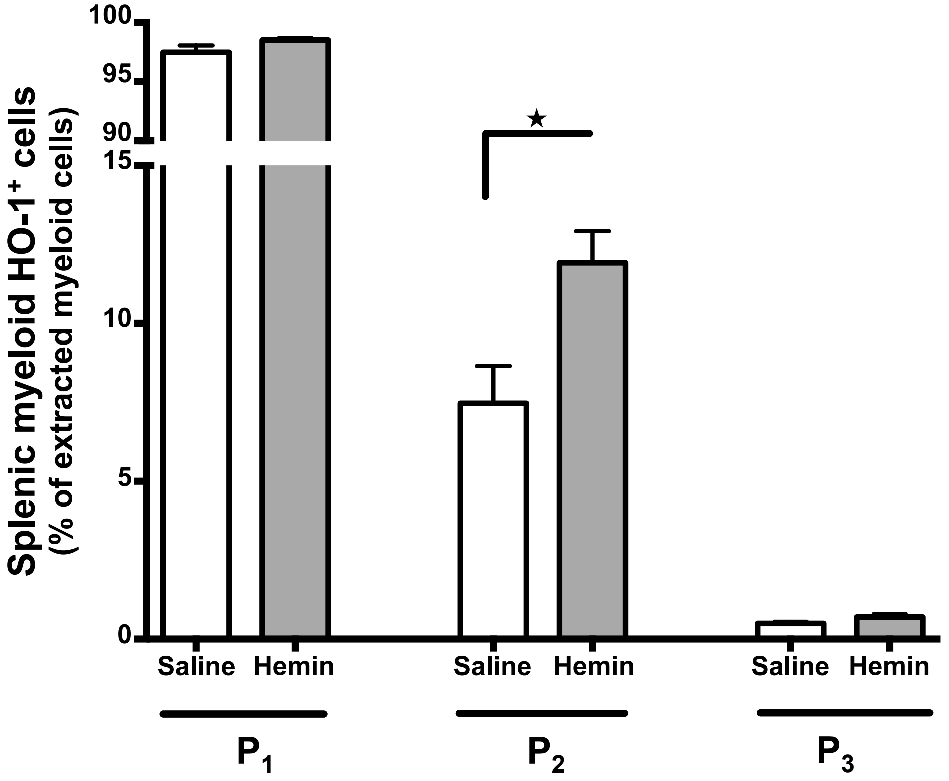

Supplemental Fig. S3. Hemin preconditioning dampens renal IRI.

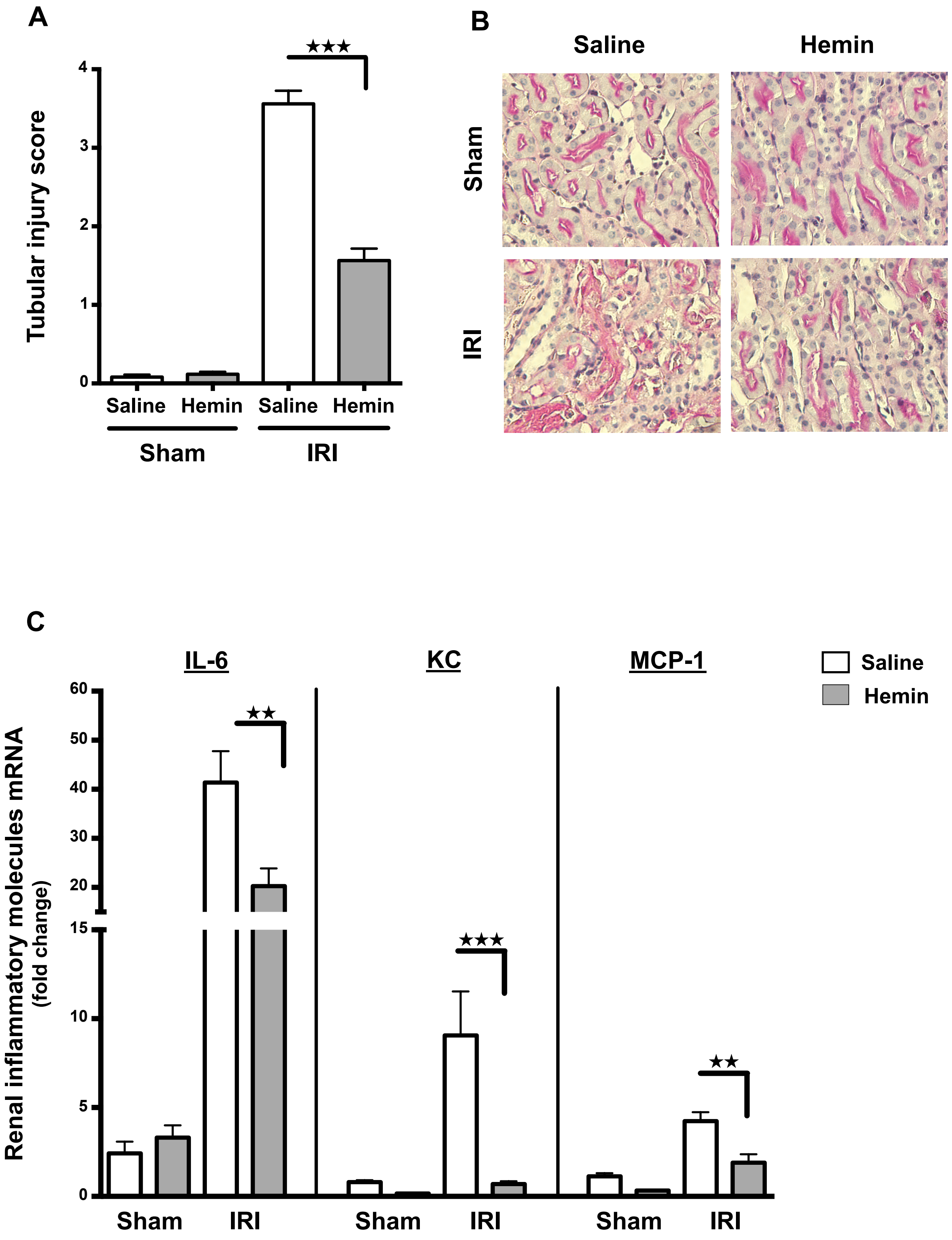

Supplemental Fig. S4. LT mice are not protected by hemin preconditioning against renal IRI.

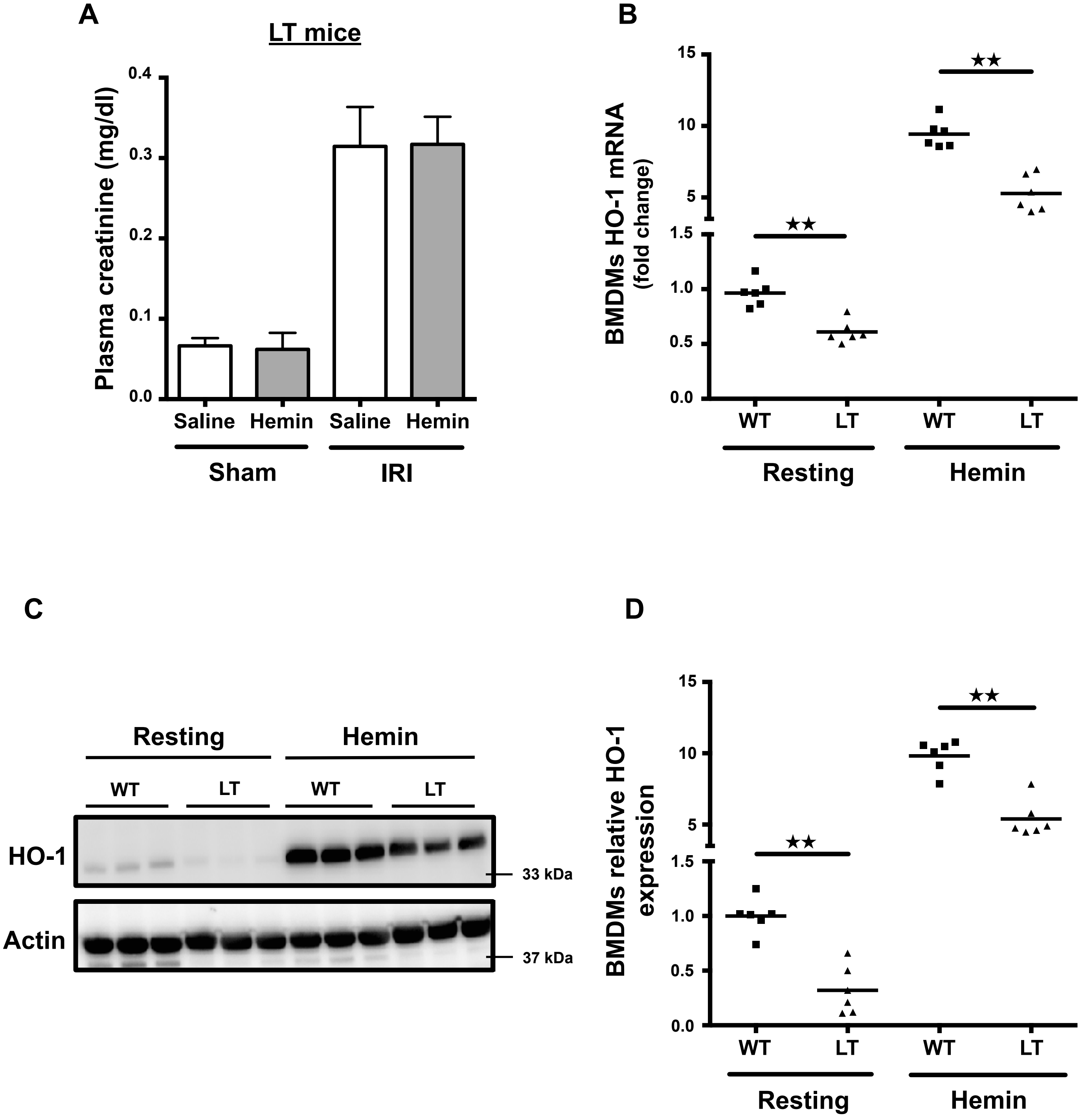

Supplemental Fig. S5. HO-1 hypomorphism is insufficient to cause particular susceptibility to renal IRI in LT compared to WT mice

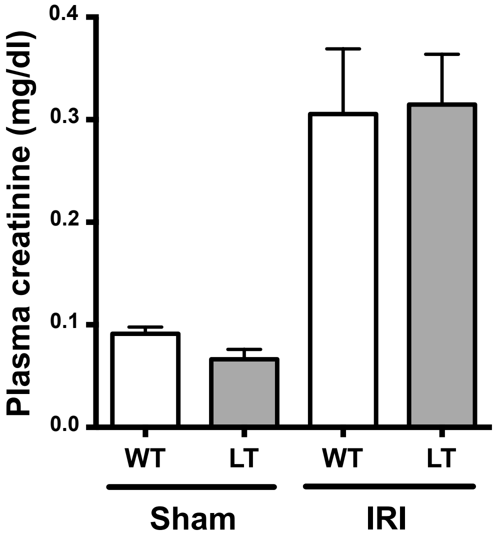

LEGENDS TO SUPPLEMENTAL FIGURES

Supplemental figure S1. Ly6G expression within renal myeloid cells. Wild-type mice (WT) underwent either bilateral renal IRI for 26 minutes or sham surgery. Twenty-four hours later, mice were sacrificed and kidneys were harvested and processed for flow cytometry analysis. Quantification of renal myeloid Ly6G^{+} cells is presented as a proportion of the myeloid cells extracted from the kidney after IRI (grey bars) or sham surgery (white bars). Results are expressed as the mean ± SEM. n=5-7 per group.

Supplemental Fig. S2. Hemin induces HO-1 expression in splenic CD11b^{+} F4/80^{lo} (P_2) myeloid cells. WT mice were treated with hemin (5 mg/kg) or saline. Twenty-four hours after intraperitoneal injection, spleens were harvested and homogenized for flow cytometry analysis. The splenic myeloid cell populations were characterized according to the expression of CD11b and F4/80 surface markers (i.e., CD11b^{-} F4/80^{+} (P_1), CD11b^{+} F4/80^{lo} (P_2), and CD11b^{hi} F4/80^{-}(P_3)). Quantification of splenic myeloid HO-1^{+} cells is presented as a proportion of the myeloid cells extracted from the spleen after hemin (grey bars) or saline (white bars). Results are expressed as the mean ± SEM, ★$p<0.05$. n=4-5 per group.

Supplemental figure S3. Hemin preconditioning dampens renal IRI. WT mice were treated with hemin (5 mg/kg, grey bars) or saline (white bars) 24 hours before sham surgery or renal IRI. At day 1 of reperfusion, mice were sacrificed. (A) Tubular injury score and (B) representative sections of corticomedullary junction (PAS-D stained). Magnification, x200. (C) Renal proinflammatory molecules mRNA expression. Results are expressed as the mean ± SEM, ★★$p<0.01$; ★★★$p<0.001$. n=16-20 for IRI groups and n=6 for sham groups.

Supplemental figure S4. LT mice are not protected by hemin preconditioning against renal IRI.
(A) LT mice were treated with hemin (5 mg/kg, grey bars) or saline (white bars) 24 hours before sham surgery or renal IRI. At day 1 of reperfusion, mice were sacrificed. Plasma creatinine levels are expressed as the mean ± SEM. n=8-9 for IRI groups and n=3-5 for sham groups. (B) qRT- PCR analysis of HO-1 mRNA levels, (C) representative images of western blot analysis and (D) quantification for HO-1 in BMDMs generated from WT (square) and LT (triangle) mice in resting and hemin stimulation (15 µM) for 24 hours. Results are expressed as the mean with scatter dot plot and representative of at least 2 independent experiments, ★★$p<0.01$. n= 6 per group.

LEGENDS TO SUPPLEMENTAL FIGURES

Supplemental figure S5. HO-1 hypomorphism is insufficient to cause particular susceptibility to renal IRI in LT compared to WT mice.
Plasma creatinine levels in WT (white bars) and LT (grey bars) mice subjected to sham surgery or 24 hours of reperfusion after renal IRI. Results are expressed as the mean ± SEM. n=9 for IRI groups and n=5 for sham groups.
